# Supplementary material for: Inactivation of genes in oxidative respiration and iron acquisition pathways in pediatric clinical isolates of Small colony variant Enterobacteriaceae
Source: Sci Rep. 2021 Apr 2;11:7457. doi: 10.1038/s41598-021-86764-4 (PMC8018945; doi:10.1038/s41598-021-86764-4)
Supplement: Supplementary file 1 — Supplementary Information [file 41598_2021_86764_MOESM1_ESM.pdf]

Supplementary Information

Inactivation of genes in oxidative respiration and iron acquisition pathways in pediatric clinical isolates of small-colony variant *Enterobacteriaceae*

Alexander L. Greninger, Amin Addetia, Yue Tao, Amanda Adler, and Xuan Qin

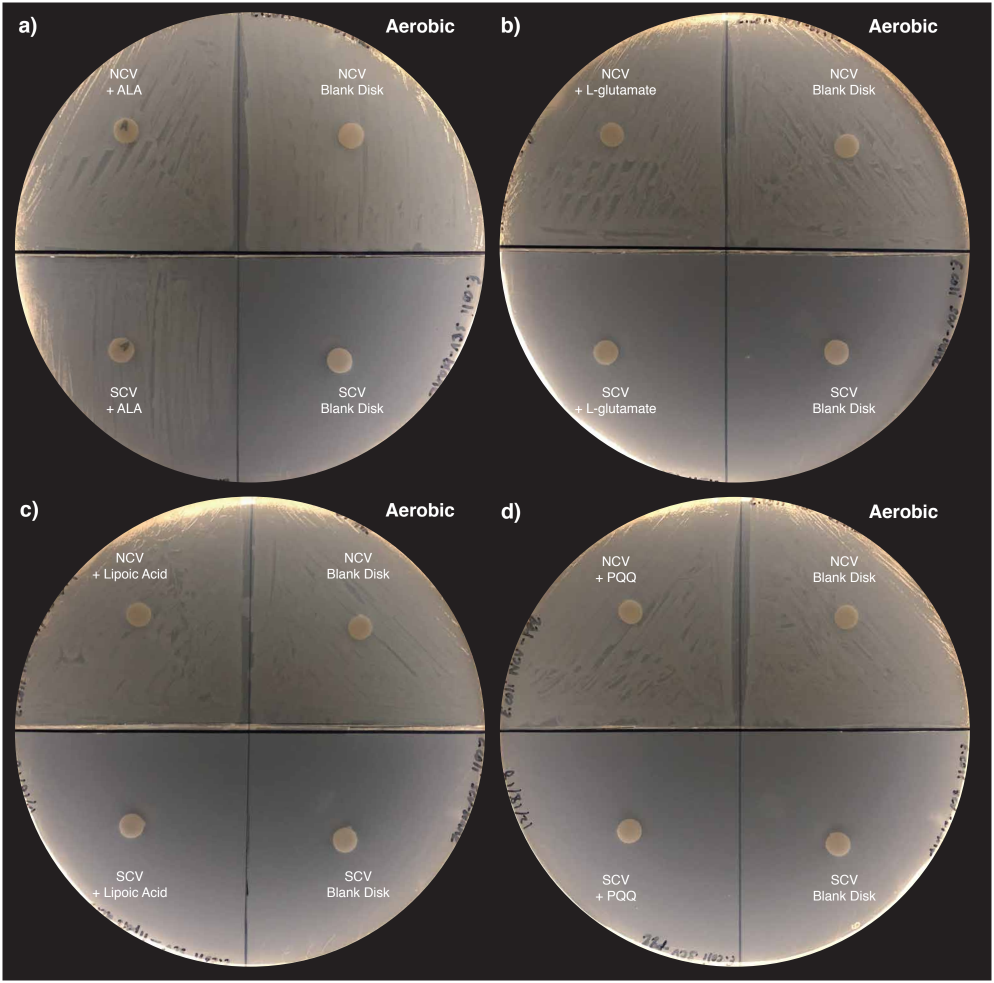

Figure S1 – Chemical rescue of Escherichia coli SCV growth was successful with δ-aminolevulinic acid (a), but not with L-glutamate (b), lipoic acid (c), or pyrroloquinoline quinone (d).

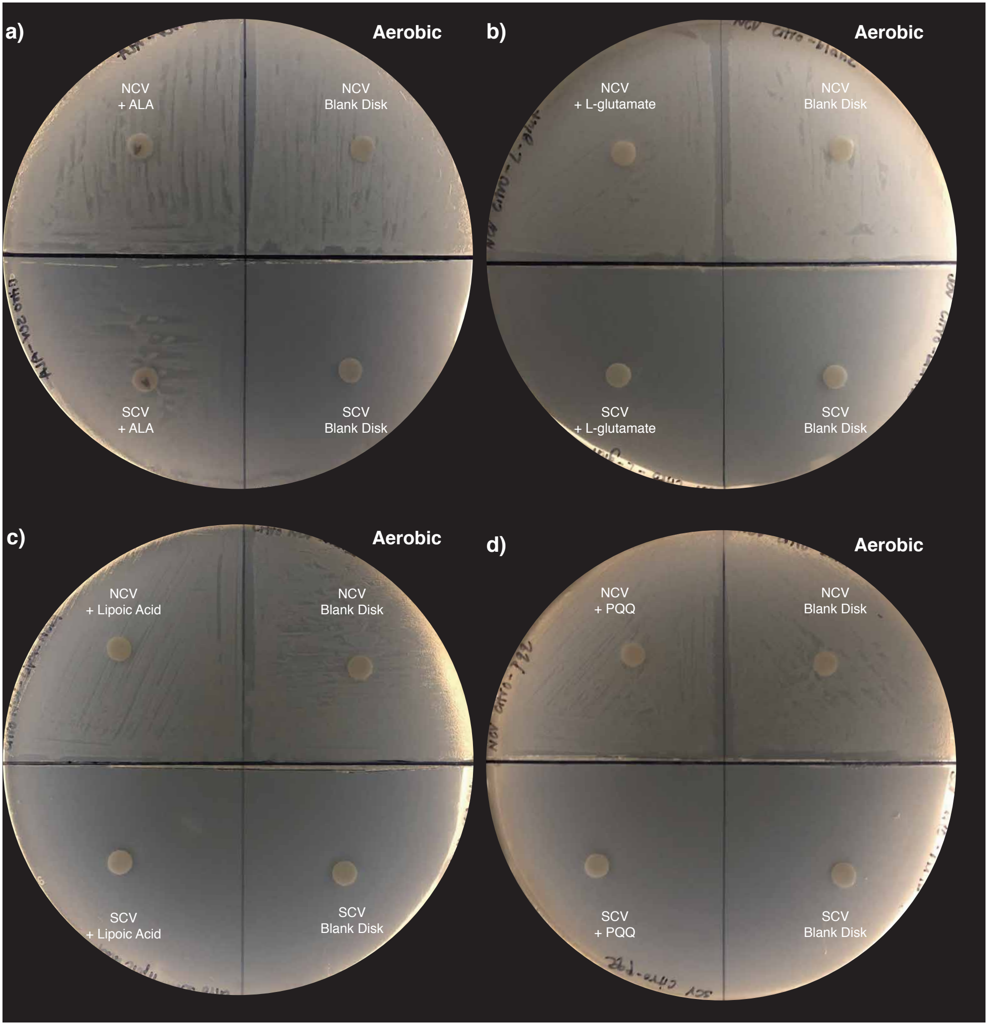

Figure S2 – Chemical rescue of Citrobacter freundii SCV growth was successful with δ-aminolevulinic acid (a), but not with L-glutamate (b), lipoic acid (c), or pyrroloquinoline quinone (d).

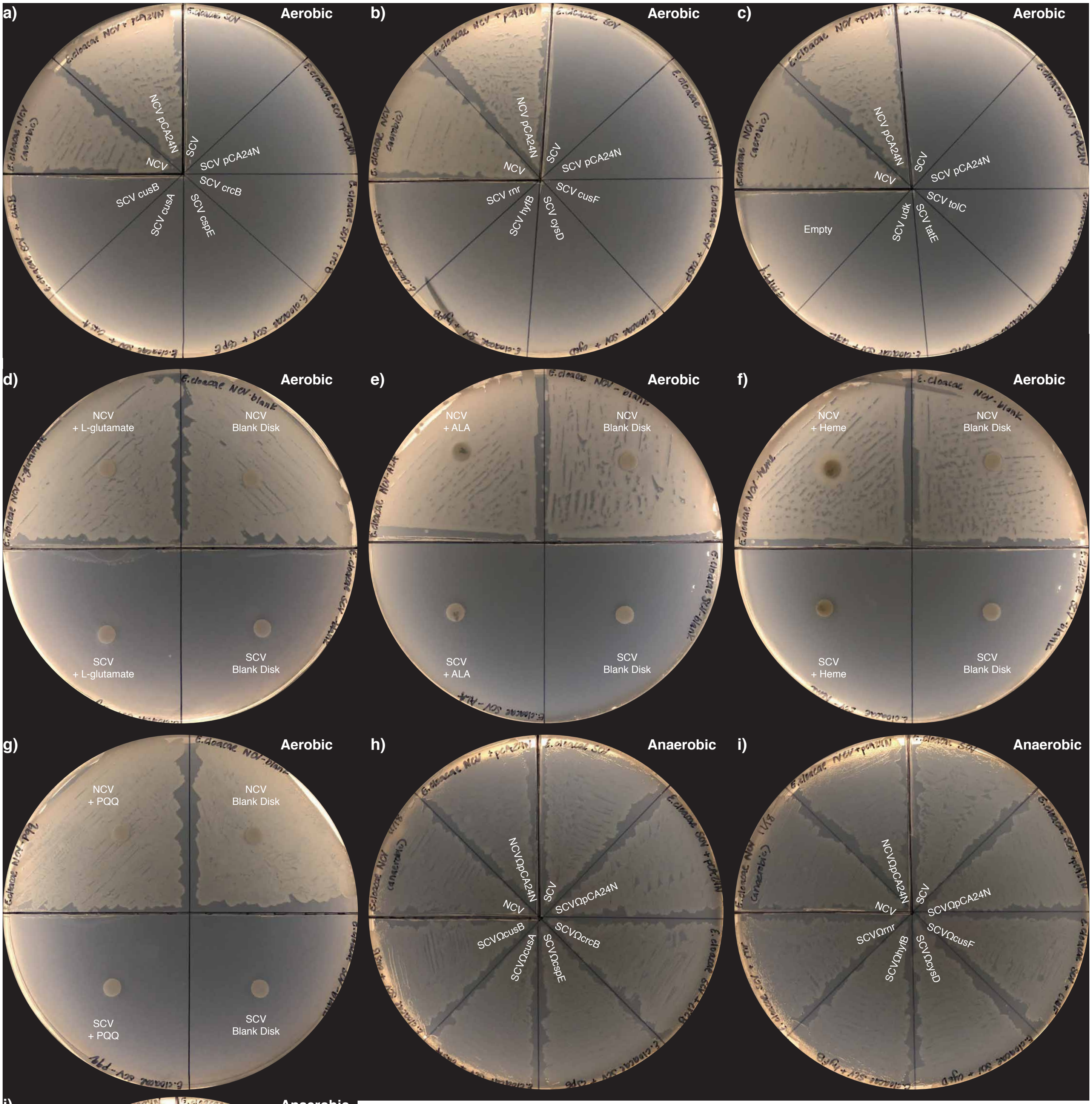

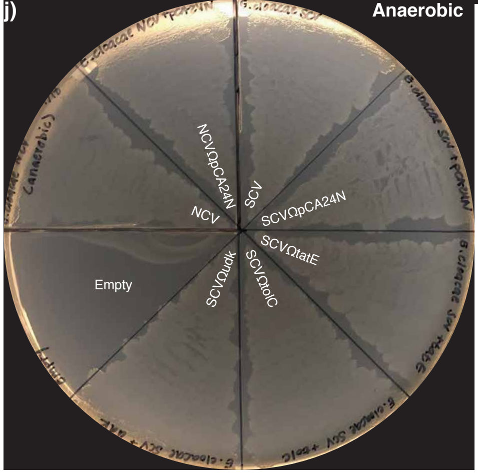

Figure S3 – Failure to rescue Enterobacter hormaechei SCV growth with deleted and mutated genes crcB, cspE, cusA, cusB, cusF, cysD, hyfB, rnr, tatE, tolC, and udk under aerobic conditions (a-c). L-glutamate (d), δ-aminolevulinic acid (e), heme (f), and pyrroloquinoline quinone (g) failed to rescue SCV growth under aerobic conditions. SCV grew similar to NCV under all anaerobic growth conditions for each of the genetic rescues attempted (h-j).
